# Uncommon Mixed Type I and II Choledochal Cyst: An Indonesian Experience

**DOI:** 10.1155/2013/821032

**Published:** 2013-05-26

**Authors:** Fransisca J. Siahaya, Toar J. M. Lalisang, Wifanto S. Jeo, Arnold B. H. Simanjuntak, Benny Philippi

**Affiliations:** Division of Digestive Surgery, Department of Surgery, Cipto Mangunkusumo Hospital, Jakarta 10430, Indonesia

## Abstract

Bile duct cyst is an uncommon disease worldwide; however, its incidence is remarkably high in Asian population, primarily in children. Nevertheless, the mixed type choledochal cysts are extremely rare especially in adults. A case report of a 20-year-old female with a history of upper abdominal pain that was diagnosed with cholecystitis with stone and who underwent laparoscopic cholecystectomy is discussed. Choledochal malformation was found intraoperatively. Magnetic resonance cholangiography (MRCP) and USG after first surgery revealed extrahepatic fusiform dilatation of the CBD; therefore, provisional diagnosis of type I choledochal cyst was made. Complete resection of the cyst was performed, and a mixed type I and II choledochal cyst was found intraoperatively. Bile duct reconstruction was carried out with Roux-en-Y hepaticojejunostomy. The mixed type I and II choledochal cysts are rare in adults, and this is the third adult case that has been reported. The mixed type can be missed on radiology imaging, and diagnosing the anomaly is only possible after a combination of imaging and intraoperative findings. Mixed type choledochal cyst classification should not be added to the existing classification since it does not affect the current operative techniques.

## 1. Introduction

Choledochal cyst (CC) is a rare congenital but not familial anomaly of either intrahepatic or extrahepatic biliary tract [1–3]. Cystic dilation may affect every part of the biliary tree, and it may occur singly or multiply. The rate is remarkably higher in Asian populations with a reported incidence of 1 in 1000, and most of the cases occurred in Japan [4]. The diagnosis of choledochal cyst is usually made in childhood (50% of cases were diagnosed in the first decade of life), and the rest might be seen later in adults (approximately 20% of cases) with symptoms related to the biliary tract pathology [2, 5]. Currently, Todani and colleagues' modified classification is the most commonly used [1, 6, 7]. This classification describes 5 types of CC. Distribution of different types of cyst varies [3, 7]. Recently, 6 cases of mixed type I and II choledochal cyst have been reported, 4 cases in children and 2 cases in adults. In all these cases, fusiform dilatation of the CBD with a diverticulum arising from the CBD was found [8, 9]. The cystic duct orifice was located on the right side of the diverticulum. We report an adult patient who had cystic dilation of the CBD (type I CC), along with a diverticulum (type II) arising from its lateral part.

## 2. Case Report

**Figure 1:**
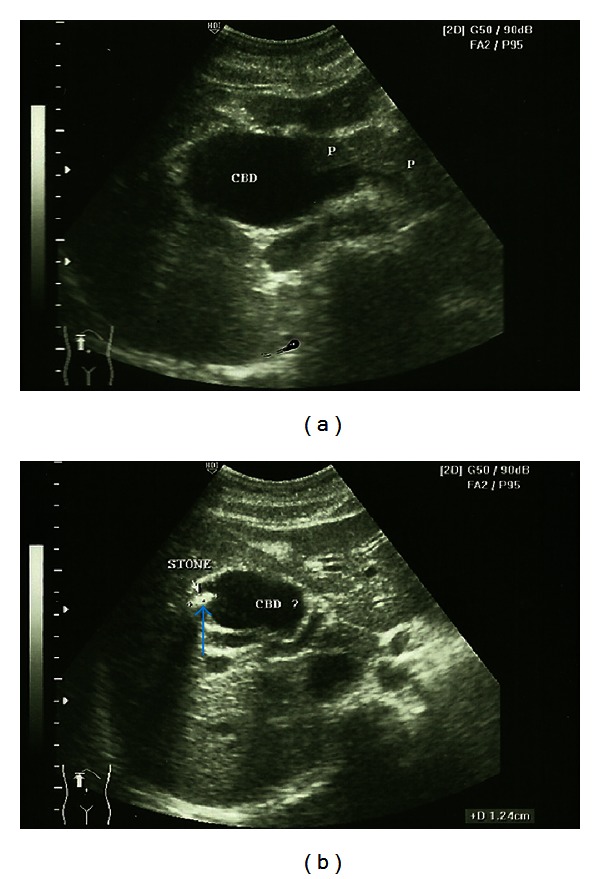
USG after the first surgery. The common bile duct was dilated, and an evidence of a stone (blue arrow) inside the dilated common bile duct was found.

**Figure 2:**
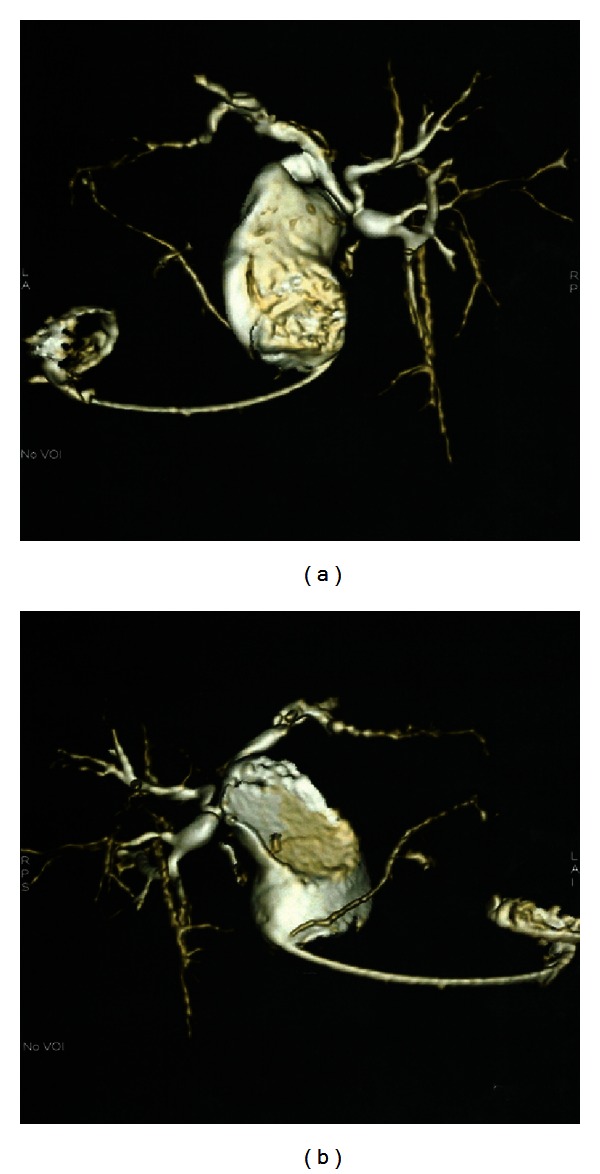
MRCP revealed extrahepatic fusiform dilation of the common bile duct without intrahepatic duct dilation, suggesting type I choledochal cyst.

A 20-year-old female complained of recurrent epigastric pain for 2 years. She was diagnosed with cholecystitis with stone and underwent laparoscopic cholecystectomy in another center. Choledochal malformation was found intraoperatively. Ultrasound (USG) and cholangiogram after laparoscopic surgery revealed extrahepatic fusiform dilation of the CBD with stone (Figures 1 and 2). Two months later ward patient came to our institution due to recurrent epigastric pain. Physical examination revealed no icterus or abdominal mass. Laboratorium examination was also within normal limits. 

Provisional diagnosis of type I choledochal cyst was made. During laparotomy, we found a diverticulum arising laterally from the middle portion of the CBD. The CBD itself was dilated in a fusiform fashion (type I CC). Complete cyst resection was performed. Based on USG, cholangiogram, and intraoperative findings, final diagnosis of mixed type I and II choledochal cyst was made. Reconstruction was performed with Roux-en-Y hepaticojejunostomy. Histopathological examination of the CC revealed no dysplasia. Three months later, the patient came for followup in good condition. 

## 3. Discussion

Diagnosis of choledochal cyst is usually made in childhood. Fifty percent of the reported cases are diagnosed in the first decade of life [3, 5]. The diagnosis is delayed in approximately 20% of cases, and these patients might be recognized first as adults with symptoms related to biliary tract pathology. 

Based on cysts' location, Todani classified the CC into 5 types (Table 1). The classification is accurate and allows preoperative planning [1, 3, 6, 7]. Distribution of the different types of choledochal cyst varies. Type I, the fusiform dilatation of common bile duct constitutes approximately 50%–80% of cases. In contrast, type II, the diverticulum of common bile duct, consists of only 2%-3% of cases [3, 4].

Mixed type I and II, the fusiform dilation of the common bile duct with a lateral diverticulum, is extremely rare. To our knowledge, only six cases have been reported, 4 in children and 2 in adults. In 2005, Kaneyama et al. reported 4 pediatric patients with mixed type I and II choledochal cyst. MRCP was useful in diagnosing 3 of these patients, and the other one was found by endoscopic retrograde cholangiography (ERCP). In 2003, Katsinelos et al. reported a similar case in a 72-year-old woman who presented with acute pancreatitis [4, 8, 9]. Another adult case was reported by Argawal et al. in 2009, a 25-year-old man who presented with recurrent abdominal pain, and diagnosis was facilitated with MRCP and intraoperative finding [8]. 

Our patient already underwent laparoscopic cholecystectomy in another center. Abdominal ultrasound and postcholecystectomy MRCP showed dilated CBD with stone. These findings suggest type I choledochal cyst. Initially we did laparoscopic investigation and then laparotomy, revealing a fusiformly dilated common bile duct with a lateral diverticulum. The main diagnostic tool for detecting choledochal cyst, especially in childhood, is ultrasonography. In adult, computer tomography is used to confirm the diagnosis; however, ERCP and MRCP are the most valuable diagnostic methods and can accurately show cystic segments of the biliary tree [1, 10].

**Figure 3:**
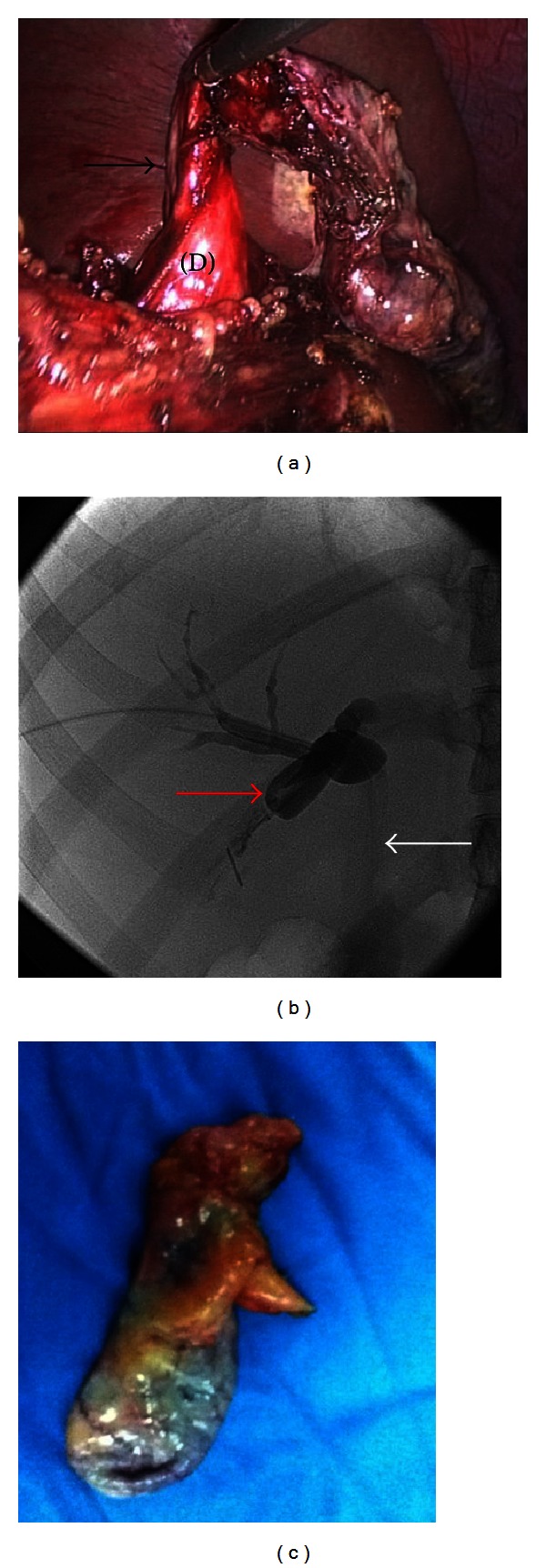
Laparoscopic cholecystectomy picture (a). The black arrow shows a narrowed cystic duct that opens directly into the diverticulum (D) before cholecystectomy. Cholangiogram (b): red arrow shows a diverticulum with end of cystic duct after cholecystectomy; white arrow shows CBD which is not filled yet with contrast. (c) Intraoperative finding.

**Figure 4:**
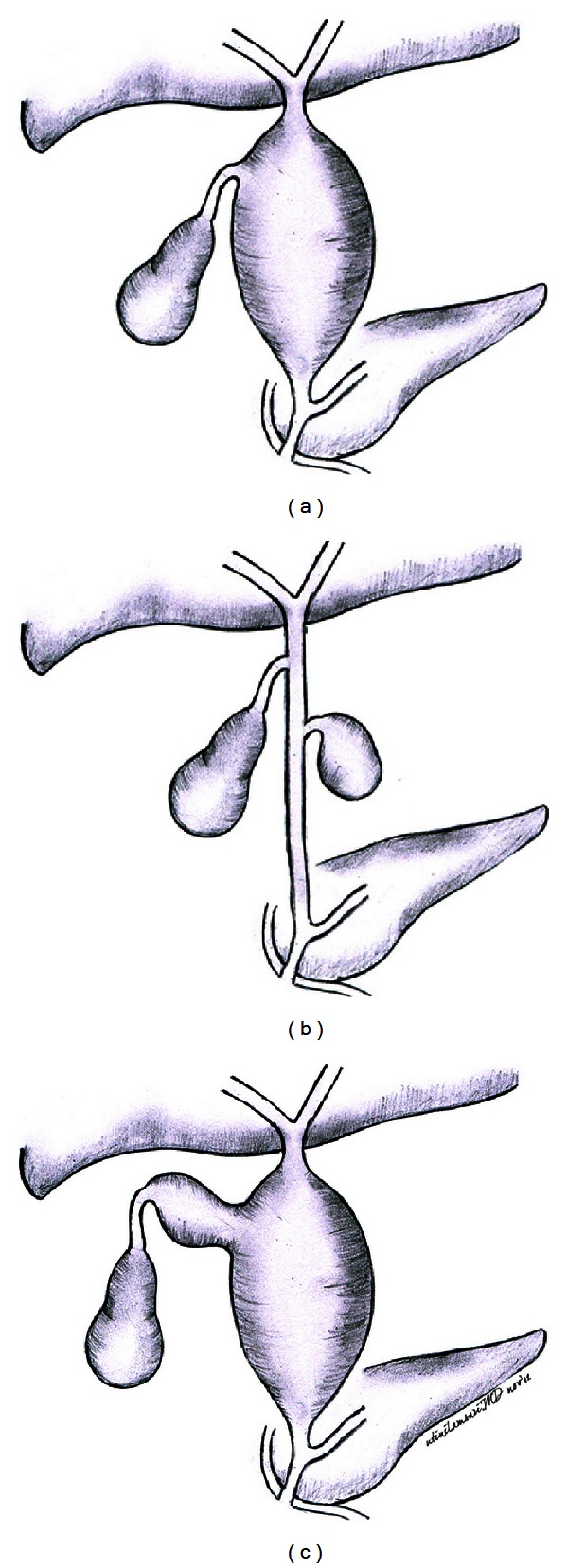
(a) Type I CC; (b) type II CC; (c) mixed type I and II CC.

We did a total resection to the choledochal cyst, followed by reconstruction with Roux-en-Y hepaticojejunostomy. To confirm the diagnosis of mixed type I and II choledochal cyst, we reviewed pictures from the previous laparoscopic cholecystectomy to identify the cystic duct (Figure 3). This is important to differ from another entity that may resemble a mixed type I and II CC which is a cystic dilation of the cystic duct associated with type I CC. This variant is differentiated from mixed type I and II CC by virtue of an identifiable cystic duct between the gallbladder and the diverticulum. Complete identification of mixed type I and II choledochal cyst by imaging is very difficult. Other authors report that diagnosing the anomaly is only possible after a combination of imaging and intraoperative findings (Figure 4) [1, 8, 10]. 

## 4. Conclusion

Mixed type choledochal cysts are rare and could be missed on imaging. Diagnosis of most cases is confirmed with either intraoperative findings or ERCP. We believe that it is not necessary to modify the existing classification to include this mixed type as a separate entity since the operative techniques are not affected.

## Figures and Tables

**Table 1:** Choledochal cyst classification.

Type	Description
Ia	Cystic dilatation of the extrahepatic duct
Ib	Focal segmental dilatation of the extrahepatic duct
Ic	Fusiform dilatation of the entire extrahepatic bile duct
II	Simple diverticula of the common bile duct
III	Choledochocele
IVa	Combine intrahepatic and extrahepatic bile duct dilatation
IVb	Multiple extrahepatic bile duct dilatation
V	Caroli disease, multiple intrahepatic duct dilatation
